# The EMBL-EBI channel

**DOI:** 10.12688/f1000research.7764.1

**Published:** 2016-01-12

**Authors:** Jo McEntyre, Ewan Birney

**Affiliations:** 1 European Molecular Biology Laboratory, European Bioinformatics Institute (EMBL- EBI), Hinxton, UK

**Keywords:** EMBL-EBI, Europe PMC

## Abstract

This editorial introduces the EMBL-EBI channel in F1000Research. The aims of the channel are to present EMBL-EBI outputs and collate research published on F1000Research contributed, in whole or in part, EMBL-EBI researchers.

## Editorial

The European Bioinformatics Institute (EMBL-EBI) has a mandate to provide freely available data and services to the global scientific community, and is a champion for open data in the life sciences. We provide a comprehensive suite of molecular databases and lead the development of Europe PMC, a free information resource offering unrestricted access to millions of full-text publications.

This *F1000Research* channel complements EMBL-EBI’s website and services, providing a new platform for research articles, but also for publishing technical documents, white papers and other materials of potential interest to the many scientists who use our resources. The flexibility of this channel allows us to share documents that may not fit neatly into the classic publishing model with more visibility and coherence: as a single collection, rather than scattered amongst various websites. We hope that this will provide an opportunity for increased transparency on the services and projects in which we participate, and make it possible to cite and refer to many kinds of useful information. In addition, we hope it will give the authors of these materials an opportunity to take credit for their work.

We look forward to contributing to a growing network of useful and forward-looking content across all *F1000Research* channels, in particular those closely related to the scope of the EMBL-EBI such as those of ELIXIR and the ISCB. We welcome your comments on the opening of this channel, and your feedback on the kinds of material you would like us to make available.

